# Supplementary material for: Different Radiation Tolerances of Ultrafine-Grained Zirconia–Magnesia Composite Ceramics with Different Grain Sizes
Source: Materials (Basel). 2019 Aug 21;12(17):2649. doi: 10.3390/ma12172649 (PMC6747583; doi:10.3390/ma12172649)
Supplement: Supplementary file 1 [file materials-12-02649-s001.pdf]

materials

MDPI

Different Radiation Tolerances of Ultrafine-Grained Zirconia–Magnesia Composite Ceramics with Different Grain Sizes

Wenjing Qin [1,2], Mengqing Hong [1,3], Yongqiang Wang [4], Jun Tang [1], Guangxu Cai [1], Ran Yin [1], Xuefeng Ruan [5], Bing Yang [5], Changzhong Jiang [1] and Feng Ren [1,*]

[1] School of Physics and Technology, Center for Ion Beam Application, Hubei Nuclear Solid Physics Key Laboratory and MOE Key Laboratory of Artificial Micro- and Nano-Structures, Wuhan University, Wuhan 430072, China.

[2] School of Physics and Electronics, Key Laboratory of Low Dimensional Quantum Structures and Quantum Control, Hunan Normal University, Changsha 410081, China.

[3] The Institute of Technological Sciences, Wuhan University, Wuhan 430072, China.

[4] Materials Science and Technology Division, Los Alamos National Laboratory, Los Alamos, NM 87545, USA.

[5] School of Power and Mechanical Engineering, Wuhan University, Wuhan 430072, China.

* Correspondence: fren@whu.edu.cn

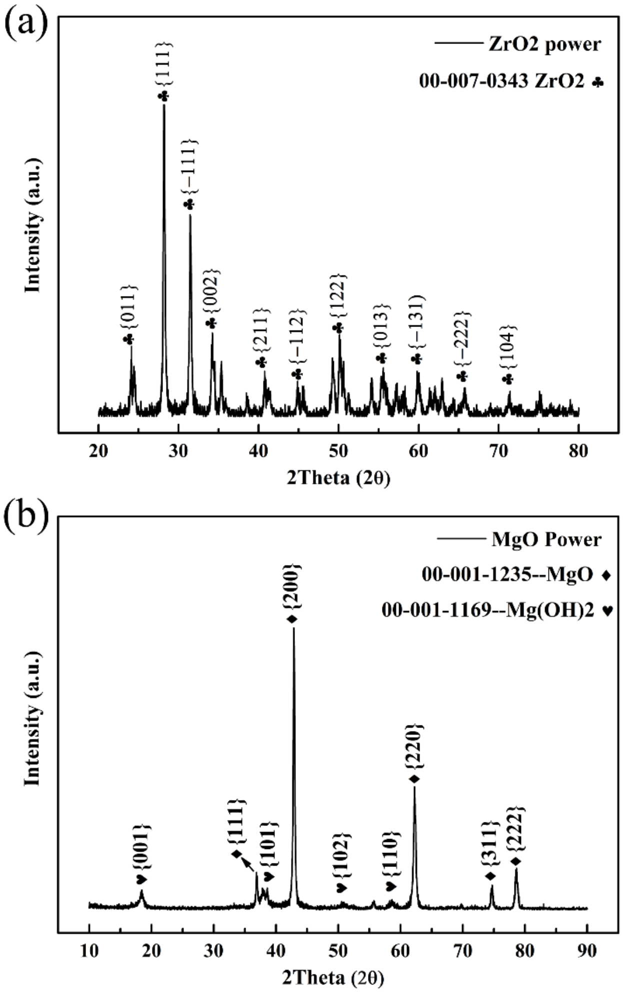

Figure S1. Grain orientations of the purchased ZrO_2 (a) and MgO (b) powders.

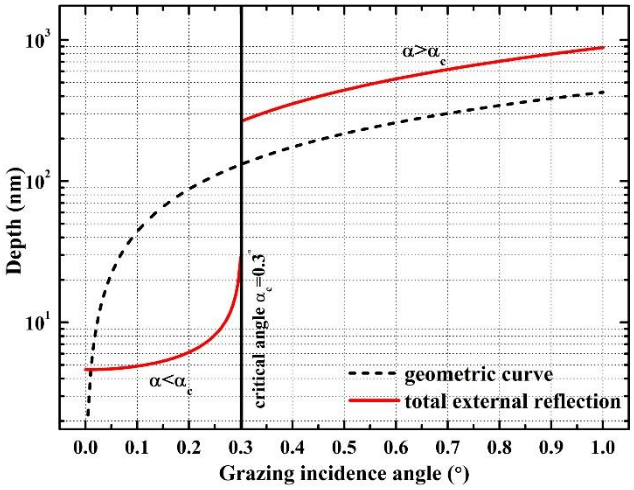

Figure S2. X-ray penetration depth in ZrO_2-MgO composite versus grazing incidence angle (α) were estimated by geometrically and total external reflection theory.

Table S1. The peak He concentration and displacement per atom (dpa) with corresponding ion range of ZrO_2-MgO, YSZ and MgO were calculated by SRIM-2013.

Sample	Density (g/cm^3)	Peak He concentration	Peak dpa
ZrO_2-MgO	4.63	282 nm—0.72 at.%	228 nm—0.21
YSZ	6.10	240 nm—0.69 at.%	198 nm—0.22
MgO	3.58	270 nm—0.83 at.%	234 nm—0.20

© 2019 by the authors. Licensee MDPI, Basel, Switzerland. This article is an open access article distributed under the terms and conditions of the Creative Commons Attribution (CC BY) license (http://creativecommons.org/licenses/by/4.0/).
